# How do mental health practitioners operationalise cultural competency in everyday practice? A qualitative analysis

**DOI:** 10.1186/s12913-018-3296-2

**Published:** 2018-06-20

**Authors:** Tooba Noor Mollah, Josefine Antoniades, Fathima Ijaza Lafeer, Bianca Brijnath

**Affiliations:** 1 Department of General Practice, School of Primary Care, Faculty of Medicine Nursing and Health Sciences, Monash University, Clayton, Australia; 2 Division of Social Gerontology, National Ageing Research Institute Ltd, PO Box 2127, Royal Melbourne Hospital, Melbourne, 3050 Australia

**Keywords:** Australia, Cultural competency, Mental health, Mental health practitioners

## Abstract

**Background:**

Despite continued policy and research emphasis to deliver culturally competent mental healthcare, there is: (1) limited evidence about what frontline practitioners consider to be culturally competent care and; (2) what helps or hinders them in delivering such care in their everyday practice. The aims of this article are to address these gaps.

**Methods:**

Qualitative in-depth interviews were conducted with 20 mental health practitioners working with immigrant patients to explore their understandings and experiences of culturally competent care. Interviews were conducted between September 2015 and February 2016 in the state of Victoria, Australia. Data were thematically analysed.

**Results:**

There were common understandings of cultural competence but its operationalisation differed by profession, health setting, locality, and years of experience; urban psychiatrists were more functional in their approach and authoritarian in their communication with patients compared to allied health staff in non-specialist mental health settings, in rural areas, with less years of experience. Different methods of operationalising cultural competence translated into complex ways of building cultural concordance with patients, also influenced by health practitioners’ own cultural background and cultural exposures. Limited access to interpreters and organisational apathy remain barriers to promoting cultural competency whereas organisational support, personal motivation, and professional resilience remain critical facilitators to sustaining cultural competency in everyday practice.

**Conclusion:**

While there is need for widespread cultural competence teaching to all mental health professionals, this training must be specific to different professional needs, health settings, and localities of practice (rural or urban). Experiential teaching at tertiary level or professional development programs may provide an avenue to improve the status quo but a ‘one-size-fits-all’ model is unlikely to work.

## Background

Mental health services in immigrant-receiving countries such as the United States (US), United Kingdom (UK), Canada, and Australia are catering to an increasingly culturally diverse population, including economic and family immigrants, refugees, asylum seekers, and undocumented immigrants. Although these culturally diverse populations have distinct mental health needs – partially because of the social, political, and historical factors shaping their migration trajectories [1] – what is consistent among these groups is an under-utilization of existing mental health services relative to the local populations [2–6]. For example, in Australia, rates of mental help seeking in immigrant communities are half the rate compared to the wider Australian community, despite the rates of mental illness being similar or potentially higher [7] with similar trends noted in the US, Canada, UK, and northern European countries [8–13].

To address this disparity concerted efforts have been made in immigrant-receiving countries for mental health services to be both economically accessible and culturally salient to immigrant communities [14–17]. Service providers are encouraged to be ‘culturally competent’ – i.e., to model “a set of congruent behaviours, attitudes, and policies that come together in a system, agency, or amongst professionals and enables that system, agency, or those professionals to work effectively in cross-cultural situations” [18]. In Australia, the construct ‘cultural competency’ has been embedded within current clinical guidelines and is seen as critical to valuing cultural diversity and tackling racism and discrimination, which represent significant barriers to adequate care [19].

However, the concept of cultural competency has also been critiqued regarding its operationalisation in everyday practice. Part of the difficulty lies in defining ‘culture’ itself as there is no consensus about what the term means [20]. Dynamic and interchanging, culture shapes the individual’s worldviews, experiences, and behaviors in relation to social interactions, religions, and environment [21]. Culture also intersects with mental health to influence symptomology, lived experience, help-seeking, treatment and management, and related physical conditions [22–31].

Difficulties around defining culture also extend to complicating understandings around cultural competency. For while a sophisticated nomenclature has evolved around the theoretical specificities of the term and its associates – e.g. ‘cultural safety’, ‘cultural humility’, ‘cultural awareness’ and ‘cultural literacy’ [32] – uncertainty remains among frontline health practitioners about what cultural competency entails in everyday practice. For example, a review of 30 community mental health centres in the US found significant variation across the centres about their understanding of what culturally competent care comprised, but only low to moderate concordance with professional definitions and standards [33]. Hwang et al. [34] describe the conundrum as: “professionals who want and need to be culturally competent are left with the message that culture matters, but continue to struggle with how to be a more culturally competent practitioner in concrete terms.” In other words, though health professionals are encouraged to be culturally competent, there is a dearth of evidence on how to achieve this. Rather, the foci has been procedural – i.e. the challenges of working with immigrant patients, whether in general practice [35–37] or specialist care [38–40]. Data is limited on the personal and everyday experiences of practitioners along the cultural continuum [18], an evidence-practice gap that risks overwhelming and isolating practitioners, many of whom already report feeling at a loss as to how to approach immigrant patients and desirous of skills-based training on how to work with patients regarding cultural influences on health and illness, diagnosis, treatment, and care trajectories [33, 35–37, 39, 41, 42]. As a first step to addressing this evidence-practice gap, this study aimed to document frontline mental health practitioners’ understandings of cultural competence, and to identify, from their perspective, what helped or hindered them to deliver culturally competent mental healthcare in their daily practice.

## Methods

A qualitative cross-sectional design was chosen as it “is multi-paradigmatic in focus,” and allows the researcher to be “committed to the natural perspective and to the interpretive understanding of human experience” [43]. This study was conducted in Victoria, the most culturally diverse state in Australia, which in turn is one of the most culturally diverse countries in the world; approximately 27% of the Australian population were born overseas and a further 43% have at least one parent born overseas [44]. Historically, Australian immigrants were from the UK and Europe but more recent arrivals are from Asia, particularly India and China [44].

Study participants included frontline mental health practitioners who had experience working with immigrant patients in the previous 12 months. Purposive sampling techniques were applied to achieve maximum variation by profession, age, gender, and location. However, we excluded General Practitioners (GPs) (also known as family physicians) from the sample because we had already undertaken similar research with this group [37]; hence it was determined that including them would not have yielded new insights.

Thirty-one potential participants, identified via websites, directories, and snowballing, were contacted through email and invited to participate. There were 23 positive responses (74.1% response rate). On further investigation, two participants were ineligible (one participant was a GP, the other did not have direct contact with immigrant patients), and one was a refugee health nurse who had to be declined as four refugee health nurses had already been interviewed. Participants came from a wide range of cultural backgrounds; half of the participants were from non-Anglo-Australian backgrounds, and half were born overseas (see Table 1). They consulted with a diverse immigrant patient population (e.g. family immigrants, refugees and asylum seekers) from established communities (e.g. Italian, Greek) to more recent arrivals (e.g. Afghanistan, Myanmar).

**Table 1:** Participant demographics

Characteristics	Counsellors (*n* = 4)	Clinicians (Psychologists/Psychiatrists) (*n* = 6)	Nurses (Refugee health/Mental health) (*n* = 5)	Social workers (*n* = 2)	Other (*n* = 3)
Age range (m)	38–53 (45.25)	28–49 (37.5)	31–57 (38.4)	24–39 (31.5)	37–58 (49.67)
Gender					
Male	0	3	0	0	1
Female	4	3	5	2	2
Ethnicity					
Anglo-Australian	2	1	4	2	0
South-American	1	0	0	0	0
Middle-Eastern	1	0	0	0	1
South-Asian	0	2	0	0	2
East-Asian	0	2	0	0	0
South-East Asian	0	1	0	0	0
West-African	0	0	1	0	0
Years lived in Australia					
Range (m)	6–38 (25.25)	9–40 (28.5)	7–38 (28.4)	24–39 (32.5)	8.5–54 (38.16)
Language other than English^a^					
Spanish	1		0	0	0
Arabic	1		0	0	1
Hindi	0	1	0	0	1
Vietnamese	0	2	0	0	0
Urdu	0	1	0	0	0
Bengali	0	1	0	0	0
Ghan	0		1	0	0
Twi	0		1	0	0
Ga	0		1	0	0
None	2	2	4	2	1
Years of experience in role					
Range (m)	8–20 (11.75)	1–15 (8.67)	2–34 (11.9)	0.5–15 (7.75)	1.5–23 (13.5)
Location^b^					
Metro (M)	3	6	2	1	2
Rural (R)	1	1	3	1	1

^a^some professionals were multi-lingual and might be counted several times

^b^some professionals work in both M and R so they maybe counted twice

Following informed consent, 20 participants were interviewed by the first author. Utilising a semi-structured guide, participants were asked about their personal understanding and attitudes to culturally competent care, support available to deliver such care, and how they would change things if they could with regards to cultural competence in mental health care (also see Table 2). Each interview was 45–60 min, audio recorded, and transcribed verbatim. Interviews were conducted in participant’s offices during business hours; consequently, participants received a $120 voucher for their time, an amount estimated to be their average hourly clinic costs if they had been seeing patients [44].

**Table 2:** Interview guide

1. What does your service provide? Who is your clientele and how diverse are they?	
2. In your own words, what is cultural competence?	
3. Do you feel that you offer culturally appropriate mental health care?	
4. Are there any systemic barriers to delivering culturally competent mental health care? *Prompt:* Funding, access to interpreters, time for consultation?	
5. Is there an attempt to evaluate what is culturally competent/culturally appropriate mental health care in your practice?	
6. What patient factors make delivering culturally appropriate mental health care easier?	
7. What patient factors make delivering culturally appropriate mental health care difficult?	
8. What kind of support do you have in regards to delivering culturally appropriate mental health care?	
9. What do you think is the attitude around cultural competence in your profession?	
10. If you could change things, what would you do to make delivering culturally appropriate mental health care in your profession easier?	

Data were analysed using thematic techniques [45] and managed in NVivo ver. 11. Data trustworthiness was ensured via input from multiple researchers to establish a coding framework, co-coding multiple transcripts independently then collectively, and constant comparison within the data and to existing literature; techniques standard to establishing rigour in qualitative analysis [46]. The Monash University Human Research Ethics Committee approved the study.

## Results

### Being culturally competent

All participants, except two, supported the need to be culturally competent and to deliver culturally competent care. The two practitioners that disagreed said that the imperative to be culturally competent risked generalisation and stereotyping, and on these grounds rejected the need to be culturally competent at all. One participant, a psychologist, viewed the term as simply a “skill set” and preferred the term “cultural intelligence” describing that as the need to be aware of each person having a unique culture. The other, a psychiatrist, viewed having any definition at all for cultural competence as counterproductive as he saw psychopathology as universal:

I think being a psychiatrist your first thing is to detect psychopathology and I think, you know, depression, psychosis, mania, sure there’s cultural variation but there is a commonality, it’s quite universal. It’s a human pathology we talk about here, so I think the rest is sort of [window] dressing really.

- *Psychiatrist#1, Male (m), 15 years work experience (ye), metro and rural.*

Among the remaining majority, being culturally competent encompassed values around flexibility, reflexive thinking, and a commitment to professional development. Flexibility referred to an ‘open’ or broad-minded approach to each patient’s cultural background; reflection to a general self-awareness of one’s own culture as influencing the therapeutic relationship; and professional development to having a ‘working’ knowledge about different ethnic groups including knowledge of languages or dialects spoken, general traditions and practices, religious beliefs, and, specific to refugee or asylum seeker populations, knowledge about political situations and conflicts.

Participants’ translation of these values into their daily practice flowed along three epistemological approaches that we classified as procedural, functional, and integrated. A procedural approach was a cautious one with a ‘right’ and ‘wrong’ way. The ‘right’ way was to first acquire facts about cultural customs or beliefs, and then to respect and work with these beliefs. The ‘wrong’ way was to ask the patient probing questions that may cause offence and/or to take offence if the patient did not adhere to Australian customs in everyday social interactions (e.g. a male patient refusing to shake hands with a female provider).

I’ve come across too many cultures to be competent in all of them… so I tend to… go cautiously, be reserved to begin with… follow their initiative, so if they’re happy to shake hands, great, I’m happy to shake hands… just being open to it, and not being offended.

- *Refugee health nurse#3, female (f), 3.5 ye, metro.*

Functional approaches focused on how culture influenced activities in daily life and on how culture mediated explanatory models of illness and disease presentation.

In the field of psychiatry… [cultural competence] is around an understanding of how a culture… influences the description of symptoms, the presentation of illness, the explanation for illness model, and the explanation for what is a diagnosis and treatment.

- *Psychiatrist#5, f, 7 ye, metro.*

Those who subscribed to a functional approach to cultural competence identified the need to build a cultural context and coupled cautious and considerate behaviour with more critical attention to different domains of cultural influence;

Cultural competence is exploring the significance say of their religion, of their ethnic group, exploring their life experiences growing up in their particular culture and their explanatory model of why they might be unwell and responding to the information they give you without preconception.

- *Psychiatrist #2, M, 11 y.e. Metro*

Integrated approaches valued diversity, preserving difference, and concentrated on increasing accessibility and integration with the wider community.

[We need to] be able to accommodate these people confidently in the community with them not having to leave their identity or their culture or their language behind.

- *Coordinator Transcultural Program#2, f, 1.5 ye, metro.*

The integrated approach placed importance on valuing diversity and preserving difference, clearly from an integrational rather than therapeutic perspective:

...cultural competence [is] how that organisation markets its business, how it works through its access for people, how it works with service delivery itself… who volunteers, who makes decisions, who’s on the board…

- *Manager, f, 23 ye, rural.*

The approach selected differed by profession, cultural exposures, ethnicity, and locality. Refugee and mental health nurses, community workers, rural participants, and those with lesspo years of experience working with immigrant communities were mainly procedural; experienced professionals often with therapeutic roles involving diagnosis and treatment (e.g. psychiatrists, counsellors, and psychologists) tended to be more functional; and participants who worked for multicultural organisations and/or held advocacy roles were largely integrative.

### Building cultural concordance

All participants recognised that building rapport and a good therapeutic relationship with their patients was critical to delivering culturally competent care. This required careful navigation of numerous interpersonal and intercultural constituents, including managing patients’ prejudices. Many female participants spoke about male patients feeling uncomfortable speaking with them or having misogynistic views that made it difficult for the patient to take their advice.

And I have to accept that, that’s not an insult, they’re not insulting me as a woman, they’re not insulting me as a person, they’re not insulting me as a practitioner, they’re just expressing the fact that it’s too shameful for them to be able to trust me in that capacity.

- *Counsellor#3, f, 20 ye, rural.*

Conversely, many practitioners from non-Anglo-Australian backgrounds felt that having an ‘ethnic’ background and speaking a second language made them more familiar and approachable to their patients.

…I had a client yesterday who said to me, “(a) you’re a woman, (b) you’re from my community so I don’t need to explain that stuff to you. You get – you’ve gotten it already without me having to talk about it. You’ve already got it”.

- *Counsellor#4, f, 8 y.e. metro*

Practitioners who had a common ethnicity with their patients further explained they felt a greater licence to question and direct their patients more strongly, explaining that a shared ethnicity was helpful in establishing a strong rapport with the patients based on their cultural moorings and expectations of health care encounters:

[The patient said] “Oh when this white lady was there she would just say, *‘Oh is that what you want?’* And then she will just give it to you.” And I [refugee health nurse] said, “*That’s why I’m dark, I’m not a white lady*.” And I know that … in our countries, if you don’t work you don’t eat, there is no money sitting somewhere to be given to you, so you have to get up and work.

- *Refugee health nurse#2, f, 34 ye, rural.*

As evidenced from this quote, some participants’ recognition of the importance of rapport did not mean they sought to equalise their relationship with their patients, preferring instead to maintain a directive approach. Some participants reported adopting an authoritative style because the stigma associated with mental illness transferred onto their professional roles and they felt that their patients did not view them as ‘credible’ compared to other health professionals such as GPs. Being more authoritative, they believed, increased their patients’ respect for them and by proxy, adherence to their treatment recommendations. Profession mediated this belief; specialists such as psychiatrists – who took a more functional approach to delivering culturally competent care – were more likely to subscribe to this belief;

Patients we see from those countries, I think there is a given kind of power differential already… so whatever the doctor says I will do that. I think there is some benefit in trying to maintain that a little bit… at least you can kind of help them with adherence… some kind of directive therapy can happen in some sense.

- *Psychiatrist#3, M, 5 ye, metro.*

We speculate that what may have also facilitated psychiatrists adopting this authoritative style was that they tended to consult with patients further along their mental health care pathway, usually after the patient had accepted the need to seek professional help for their distress. In contrast, allied health staff such as social workers and refugee health nurses, who consulted patients earlier in their care-seeking journey, were much more wary of how they broached the topic of mental illness and the language used. Practitioners who consulted with patients in a general health setting (as opposed to a specialist mental health one) said they never used psychiatric terminology or labels, or only did so incrementally over time.

You never label something to start with. You build it up, you describe things. So, for depression, example, it might be, you know, feeling really tired and not wanting to get out of bed and not enjoying the things you used to enjoy and, you know, feeling a bit irritable… down the track you get to the point where you say, “Well, you know, like, a word that we use for that is ‘depression’”.

- *Social worker#1, f, 0.5 ye, metro.*

### Tackling language barriers

Clear communication was integral to delivering culturally competent care and participants reported that being multilingual, having access to multilingual colleagues, and/or access and capacity to use interpreter services was fundamental to fulfilling their role. However, using language services was also seen as time consuming, labour intensive and at times inaccurate, sometimes producing unsatisfactory results through misinterpretation and difficulties communicating with the interpreter. Such frustrations were most strongly voiced by psychiatrists, who usually had strict consultation times and high patient load.

Sometimes it does feel unsatisfactory that I’m not sure that I’m being helpful or not sure that the communication has gotten across. I’m not sure whether it be because of me or because of the interpreter or because of the patient, all three factors….

- *Psychiatrist#5, f, 7 ye, metro.*

I suppose I do my best… don’t show too much emotion, don’t show the frustration because [consultation using interpreter] takes twice as long and… they don’t answer what you want….

- *Psychiatrist#1, m, 15 ye, metro and rural.*

Participants in rural and regional communities also reported feeling under-resourced with regards to accessing face-to-face interpreters and relied more on telephone services, even though the former communication was preferred. Additionally, rural practitioners voiced that many professionals in their community were inexperienced, not adept at using language services, and were apprehensive to do so.

There seems to be a lot of nervousness with staff around using language services in particular. And so I think sometimes they dismiss the importance of it by saying well, you know, they can get a child to translate for somebody and those kinds of things. Which is really interesting because we wouldn’t have the expectation that we would be getting other people’s children to provide sensitive psychiatric information for them.

- *Social worker#2, f, 15 ye, rural.*

Such attitudes were also reported in urban settings by several practitioners who said that mainstream services lacked adequate language resources, and had confusing and cumbersome processes, which limited immigrant patients’ access to these services. One practitioner commented that she had received letters from specialists asking for immigrant patients with low English proficiency to bring family members to interpret the consultation.

*I’ve seen letters have come from private psychologists and other specialist services, “Can you bring in a family member to translate for you?” in the actual letter…How are you making [an] assessment if you’ve not got the communication pathways open?*

- *Counsellor#4, f, 8 ye, metro.*

While difficulties around accessing and using interpreter services were predominantly discussed from the individual practitioner and organisational perspective, many practitioners reflected that the Western model of health care itself was a cultural construct that was confronting, rigid, and inflexible, thereby making it difficult to work with patients who came from outside this medical paradigm. Overall, participants felt that there was a widespread societal lack of appreciation and knowledge of other cultures by Australians, which also included those working within the healthcare sector. According to them, reversing this trend requires broad educational intervention from primary school to tertiary education and professional development.

All of us have particular expectations and conceptions of how people should behave when they’re depressed and what treatment they should get… particularly say if you’re training in the Western model and it’s like, ‘Do this, prescribe this, prescribe CBT [cognitive behaviour therapy]... in that set way.’ So as a psychotherapist myself, biggest challenge in working in a culturally responsive way… to put that [assumptions and conceptions] aside without imposing it on the patient….

- *Psychiatrist#2, m, 11 ye, metro.*

### Institutionalising cultural competence

Participants said organisational attitude and a culture of valuing diversity needed to be developed in a top down manner, displayed from management, and encouraged among frontline workers including practitioners, administration, and support staff. The value placed on cultural diversity was seen to be reflected in its predominance within the organisation such as whether cultural diversity was an agenda item in meetings, exemplified by management staff participating in cultural events, and if staff were provided with culturally relevant education and resources. The importance of cultural diversity was also reflected in the diversity of staff hired within the organisation and whether that reflected the diversity of the community, including having bilingual or multilingual workers to improve access for immigrant patients. All staff, including reception staff, being offered training for cultural competence was also seen by participants as institutionalising cultural competence. One participant, a director of a rural multicultural service, commented that she personally saw that all her staff were adequately trained in using interpreter services. Some of these organisations displayed commitment to improvement through using staff and patient feedback and evaluation.

The organisation provides a lot of avenues for people to do professional development training if they want to… they really champion professional development… which really helps.

- *Counsellor#4, f, 8 ye, metro.*

Collaboration with external organisations and services were seen as important to patient care as often immigrant patients had multiple and varied issues. Similarly, community liaison was also seen as crucial because it helped break down the stigmatising barrier of mental health care, put a face to an organisation, and brokered stronger therapeutic relationships between local immigrant communities and practitioners. For some participants, creating good community bonds was important to build their credibility and reputation as a practitioner, and they valued the brokering of these relationships at the individual and organisational levels.

…It’s very important. Our CEO for example he has contact with community leaders. There is contact with community leaders in the community here in [organisation] so the manager has those contacts. Consultation with the community, that happens as well.

- *Counsellor#2, f, 9 ye, metro.*

Conversely not all organisations that participants worked in were supportive and barriers to culturally competent care included being under-resourced, colleagues holding prejudiced attitudes, and being inexperienced. The inexperience and apprehension described among some rural practitioners stemmed from lack of exposure to immigrant groups in their community. Lack of exposure, in turn, made it difficult to change social prejudices and community attitudes. One rural practitioner felt that some of her colleagues were in denial about existing health disparities in immigrant communities and saw specialised services as a ‘privilege’ rather than a way to promote health equity and access.

You still do hear people talk about - if there’s a specifically targeted or funded program or service that might be refugee health or it might be for Indigenous consumers that it can sometimes… can see that as unfair, why is this group getting something that everybody else isn’t getting? Which again sort of misses out that idea that well part of the reason that they are is to try and equal the playing field.

- *Social worker#2, f, 15 ye, rural.*

### Sustaining cultural competence

Personal motivators and professional resilience were the cornerstones to developing and sustaining cultural competency. All participants were intrinsically motivated to work with immigrant patients and most felt a connection and empathised with immigrant patients. Positive experiences with different cultures during childhood, living or working overseas, being raised with strong ‘values’ of universality and equality for all, and having strong connections to multicultural communities with a desire to ‘give back’ were common personal motivators. Professional motivators for participants included working alongside colleagues that shared a similar worldview and saw their work as ‘more than a job’.

And also the staff that are there because they’re passionate about those issues for starters… we’re there because we care and this is where we want to be. Like this is what interests us and what makes us tick… It’s not just a pay cheque.

- *Refugee health nurse#1, f, 2 ye, metro.*

For participants who worked with refugee and asylum seeker communities, social justice was an additional powerful motivator. The volatility of the Australian government’s immigration policies, involuntary long-term detention, and indeterminate visa status were highlighted as structural factors that perpetuated mental illness among refugee patients. While many participants expressed helplessness and frustration at not being able to influence immigration policy and practice, they did view their work as political and subversive:

One of my colleagues said to me, “I know I’m getting paid for this, but I almost feel like doing this job is a political act of defiance in itself.” This is when Abbot [conservative Prime Minister] was in power… I think I agree.

- *Refugee health nurse#1, f, 2ye, metro.*

Personal motivation was considered to be an important buffer to the emotional and professional toll of providing culturally competent mental healthcare. Participants recognised that their work was intense and delivered in a context of resource scarcity, resulting in high workload and demands on time. Among counsellors, working with refugee and asylum seeker populations, there was also often a significant emotional toll that emanated from listening to and working with patients who had experienced torture and trauma.

I can feel the pain quite intensively sometimes. I can feel pain. There’s been lots of times where I just left that counselling room and I’ve gone to the toilets and cried … So I try to put them on my case notes and I leave it behind because the stories we hear are really horrible … So yes, there is quite a lot of heaviness that we carry and being aware again of how it impacts on you is - is really my duty to take care of myself.

- *Counsellor#2, f, 9ye, metro.*

For participants these work conditions underscored the need to be self-aware, and regulate emotions. Offsetting the toll of working in transcultural mental health, many practitioners stated that they felt satisfaction working in the area because they felt they were helping those who genuinely needed it. Practitioners also cited that many immigrant patients were more grateful and sincere than some Anglo-Australian patients, which was personally motivating for them. There was also a sense of satisfaction in seeing people develop and recover and therefore feeling useful and valued as a professional, a view strongly held by refugee health nurses:

There are days when I feel like I’m banging my head against a wall and I just “My God, if one more person is late, I’m just going to...” but then there’s days when some beautiful woman, who has had a tragic life and is still not safe, is nonetheless pleased to see me and gives me a hug, and thinks that I’m great.

- *Refugee health nurse#3, f, 3.5 ye, metro.*

## Discussion

This study investigated frontline mental health practitioners’ understandings of cultural competence, and identified, from their perspective, what helped or hindered them to deliver culturally competent mental healthcare in their daily practice. To date there has been limited exploration of individual practitioners’ understanding and delivery of cultural competence [35, 37, 47–50], which limits the provision of knowledge, resources and support to frontline providers to better enable them to treat immigrant patients with mental health conditions. In response to previous calls to explore cultural competence across diverse professions, settings, and localities [51], our work reveal four key findings: (1) while conceptually there are some common understandings of what cultural competence includes, its operationalisation differs by epistemology and sociological diversity; (2) these differences can translate into complex ways of building cultural concordance with patients; (3) the reliable use of interpreters and organisational apathy remain major barriers to promoting cultural competency; and (4) organisational support, personal motivation, and professional resilience remain critical facilitators to sustaining cultural competency in everyday practice. We elaborate on these findings below.

Common values associated with being culturally competent included flexibility, reflexive thinking, and a commitment to professional development; values described in previous studies [40, 52, 53]. However, while practitioners were able to identify these values, there was significant variation in their operationalisation. Procedural, functional, and integrative approaches were three typologies to emerge from our analysis; such typologies differed by profession, health setting, locality, and years of experience. Participants who worked in non-specialist health settings (e.g. community care), in rural areas, with less years of experience working with immigrant patients were mainly procedural in their approach. In contrast, experienced professionals, working in psychiatric care, and in urban settings tended to be more functional.

Practitioners who were more functionally-oriented, also tended to be more authoritative in their communication with patients justifying this approach on account of mental illness stigma, language barriers, and different explanatory models of illness; patient factors that resonate with previous research [54–58]. However, it is important to note that just because some participants’ recognised the importance of rapport, did not mean they sought to democratise their relationship with their patients. Instead, as some of our data show, they actively sought to maintain a power differential, perceiving in some instances that this was an expectation from their patients and might be beneficial to their therapeutic alliance. We are unable to comment on whether such perceptions manifested from clinical experience, or reflected implicit biases, or what impact this had on clinical practice and patient outcomes. These are important avenues for future inquiry. Our data do reveal that practitioner’s communication style and ways of building cultural concordance was influenced by their own ethnicity and where they worked along the health-seeking continuum. Practitioners dealing with patients when they might be commencing their care-seeking journey were much more cautious and equivocal as compared to specialists who consulted with patients further along this continuum.

Similarly, those who leveraged their culturally diverse background and/or cross-cultural experience in building rapport with their patients seemed much more confident in negotiating the boundary between patients’ cultures and the health system than those who felt limited by their ethnicity and gender, the latter most likely to be Anglo-Australian women. Further, as highlighted by some practitioners, cultural mores relating to the social position of women in some cultures, may inhibit the acceptability and uptake of their care provided by female practitioners to conservative male patients. However, research with Indian-Australian patients with depression suggest that neither culture, gender nor age affect the therapeutic relationship. Instead mutual understanding and rapport appear to be more important [59]. Whether this applies in other immigrant communities would require further investigation.

Among those participants who felt they were building cultural concordance, many of the communication styles described tended to homogenise ethnic differences between practitioner and patient but highlighted ethnic differences from the mainstream community (e.g. “I’m not a white lady”). Historically, research has shown that patients prefer for their health practitioners to be from the same cultural background as their own, especially for those with language barriers [58]. In such instances, the practitioner is a ‘boundary crosser’, part of the same culture as well as part of the health sector, trusted by both groups [60]. However, in this study there was little (if any) direct ‘matching’ between diverse practitioner and patient cultures. Rather, practitioners highlighted patient differences from the mainstream in order to act as a boundary crosser between multicultural Australia and a biomedicalised health sector. Such tactics were noteworthy because they offer insight into how culturally diverse workforces are adapting to culturally diverse patient populations vis-à-vis building cultural concordance within a western biomedical paradigm. Following on previous work that emphasises the importance of the clinician’s interpersonal skills to overcome cultural communication barriers [40, 61], we call for more textured analyses to investigate the effects of these different styles of communication on therapeutic relationships, treatment adherence, and patient outcomes.

Consistent with existing literature [3, 57, 61–64] language was also identified in our study as a barrier to delivering care; being bi- or multi-lingual, having access to such colleagues, and/or using interpreter services were critical to overcoming language barriers. By contrast, language was not identified as a barrier in our research with GPs [37] partially because some of the GPs interviewed were either multilingual and/or relied on family members to interpret during the consultation. While none of the participants in this study used family members as interpreters, this problem has been widely noted as an impediment to effective consultation [65–67]. Other barriers associated with interpreters included the accuracy of the interpretation and the time taken to interpret, barriers also identified by Huang and Phillips [68].

The sustainability of culturally competent care was felt to be negatively affected by systemic factors, such as inflexibility among mainstream services and certain health and immigration policies. Some practitioners felt that cultural competence was ignored or trivialised on a wider scale and that some of their colleagues harboured prejudiced and discriminatory attitudes. This was particularly noted in rural areas, which could be a result of limited exposure to immigrant communities [69]. The greatest enablers identified for culturally competent care among participants were organisational supports and personal motivation. There is significant literature exploring the importance and need of organisational support and commitment to operationalise cultural competence [70, 71]. Concordant with the literature [70, 72–74], participants found especially valuable an organisation and staff that were respectful and valued diversity, networked with other organisations, nurtured community liaison and integrated these components into everyday practice.

Personal motivating factors resulting from positive personal experiences of culture, social justice values, genuine interest in culture and differences helped practitioners overcome the high work demand and emotional toll of working in this area. While vicarious trauma is an area increasingly being explored among frontline mental health staff who confront violence and abuse in their daily work [75–80] the emotional toll of working with refugee and asylum seekers in immigrant-receiving countries such as Australia is not well documented. Neither are the emotional effects of continuously dealing with punitive government immigration policies and its likely impact on staff turnover in the sector. To reduce vicarious trauma it is crucial to first recognise the phenomenon, then for practitioners to talk to and debrief with people they feel connected to and supported by [81]. Greater support is needed for mental health workers, especially those who work directly with victims of torture or trauma, as well as rural practitioners and communities, given government pressure on new refugees and immigrants to settle in these area to reduce pressure on metropolitan cities [82]. Additionally, there needs to be an examination of the attitudes and prejudices that exist within the wider medical community as mental health professionals do not work in isolation and are affected by the views and potential stigma of those around them.

Limitations of our study include selection bias as it may have captured views from those particularly interested or passionate about the topic rather than other practitioners in the field. There may have also been an element of social desirability bias among participants and they could have presented views of cultural competence as being more important than they personally believed, although this did not stop some practitioners from expressing views of cultural competence as redundant. Our cohort was reflective of the workforce in question [83], but more female participants were interviewed which influences the generalisability of the findings. Finally, this study reported findings of self-perceived cultural competence, which might not be reflective of practices of cultural competence overall. The strengths of the work was its focus on the individual and personal experiences of mental health practitioners, including a range of frontline providers, in rural and metropolitan settings, in a multicultural state, which increases the generalisability of the results.

## Conclusion

The unprecedented scale and scope of contemporary migration, whether by economic and family immigrants, refugees, asylum seekers, and/or undocumented immigrants (among others), require that we continuously query how frontline mental health practitioners understand cultural competence and operationalise it in their everyday practice. By doing so, this study adds to the under-researched area of culturally competent mental healthcare practice. It provides insight into the perspectives of those working on the frontline, especially the near-silent voices of rural practitioners, thus giving a deeper and richer understanding of the challenges faced by mental health practitioners and future directions for research and training. This research suggests that understanding and conceptualisation of cultural competence is inconsistent across the board, influenced not only by knowledge and awareness of the issues but also by locality, profession, work setting, and gender among other factors. Taking these different factors into consideration there needs to be a greater focus on developing multi-faceted interventions to inculcate cultural competence across the medical professions. We strongly counsel against a ‘cookie-cutter’ approach; not only is this counterintuitive to the underlying philosophy of cultural competency, but also the evidence shows no to only moderate effect with such training [84]. Rather, the focus should be on catering such interventions to the needs of different professions in different settings, accompanied by thorough evaluations of efficacy.

## Abbreviation

GP: General Practitioners
